# Supplementary material for: Are carbon emissions trading and green financial instruments synergistic? -Comprehensive quantitative research based on content analysis
Source: PLoS One. 2024 Mar 7;19(3):e0298601. doi: 10.1371/journal.pone.0298601 (PMC10919601; doi:10.1371/journal.pone.0298601)
Supplement: S1 Data — (ZIP) [file pone.0298601.s001.zip › Data/地级市Z_F工作报告.zip!/║╙▒▒/▒ú╢¿╩╨/└┤╫╘╣1⁄2╓┌║┼í╛┬φ┐╦╩2╛▌═°í┐_2016.docx]

各位代表：

从2016年开始，我们已进入第十三个五年规划时期。根据市委十届十次全会通过的《关于制定国民经济和社会发展第十三个五年规划的建议》，市政府制定了《保定市国民经济和社会发展第十三个五年规划纲要(草案)》。现在，我代表市人民政府向大会作工作报告，请连同《纲要(草案)》一并审议，并请市政协委员和列席会议的同志提出意见。

一、“十二五”时期经济社会发展回顾

“十二五”时期是我市积极应对严峻复杂形势、经济持续健康发展、城乡面貌明显变化、人民生活不断改善的五年。特别是党的十八大以来，在省委省政府和市委坚强领导下，在人大法律监督、工作监督和政协民主监督下，我们积极认识、主动适应、注重引领经济发展新常态，统筹推进稳增长、促改革、调结构、抓协同、治污染、惠民生各项工作，拼搏进取、奋力前行，较好完成“十二五”主要目标任务，为全面建成小康社会奠定了坚实基础。

综合实力大幅提升。到“十二五”末的2015年，全市生产总值达到3000.3亿元，五年年均增长9.1%(全省平均8.5%);人均生产总值达到2.9万元，年均增长8.3%(全省平均7.6%);一般公共预算收入196.9亿元，年均增长18.5%(全省平均14.7%)。规模以上工业增加值986.8亿元，年均增长10.6%(全省平均 9.7%)。累计固定资产投资9984.6亿元，年均增长16.5%。三次产业结构调整为11.8∶50.0∶38.2。服务业增加值达到1146.1亿元，年均增长10.1%，占生产总值的比重提高了3.5个百分点。农业稳定发展，粮食生产“十二连丰”，农业产业化经营率达到66.6%。2015年年初确定的24项计划指标，有22项超额完成。其中，生产总值较上年增长7%，全部财政收入增长9.0%，一般公共财政预算收入增长10.6%，城乡居民人均可支配收入分别增长8.8%、10.3%360小说。

协同发展成效初显。《京津冀协同发展规划纲要》把保定纳入“中部核心功能区”，列为“区域性中心城市”，把京保石发展轴作为三大发展轴重点打造，明确提出“推动京津保地区率先联动发展”，在顶层设计上确立了保定的战略地位。京石高铁、津保城际建成通车，张石、保阜等高速全线通车，京昆高速与北京顺利贯通，京港澳高速改扩建工程全面完成，京津保地区1小时交通圈基本形成，京津冀交通一卡通在我市率先推行。我市与京津合作项目达248项。高新区和徐水大王店汽车产业园跻身千亿级园区，总投资1000亿元的保定汽车科技城项目、总投资382亿元的长城汽车整车及零部件项目、总投资128亿元的涞水电子科技产业园、总投资100亿元的中汽零保定产业基地等加快建设。保定·中关村创新中心作为中关村在京外设立的首家创新中心，现已入驻企业42家;白洋淀科技城总投资313亿元的13个项目集中签约，8个项目开工建设;北京林业大学白洋淀生态研究院、北京服装学院容城服装文化创意中心正式揭牌，北京儿童医院成功托管保定儿童医院。与36家央企集团签署了总投资1665亿元的75项合作协议，与150家北京文化、教育、卫生机构开展对接合作。

创新发展活力迸发。坚持创新驱动、高端引领，积极培育创新主体、聚集创新要素、搭建创新平台，创新发展亮点纷呈。高端创新平台不断涌现。长城汽车新技术中心、涿州国家农业高新技术产业示范区、高碑店国家建筑节能技术国际创新园等加速推进，清华启迪之星创业孵化器、微软云暨移动应用孵化平台项目落户我市。高新技术产业快速发展。高新技术企业达到185家，科技型中小企业达到4151家，均居全省第二。“河北省科技进步奖”获奖总数连续四年居全省第一。院士工作站达到18家，国家、省、市各级重点实验室、工程技术中心、产业研究院等达到169家。协同创新共同体成效显现。保定科技金融创新中心暨京津保创新创业大街启动，与中国科协、中国科学院、中关村、清华大学、北京大学等开展全面战略合作，组建产业技术创新联盟20个，建立全国学会服务站55个，筹建了大学生创新创业联合会，被中国科协命名为首个“全国创新驱动发展示范市”。

城乡面貌明显改善。拉大城市框架，优化城市布局，提高辐射带动能力，城乡统筹发展日新月异。清苑、满城、徐水撤县设区，主城区面积扩大8倍，市区人口由119万增加到280万，“大保定”格局正在形成。市区交通网络日趋完善，能源、水利、环保等基础设施项目建设有序推进。清真寺、西大园、府河三大片区改造即将完成。主城区商业服务设施更加完善，万博广场、茂业中心、华创广场、未来石等城市综合体全部建成。城市管理“6S”行动持续推进，市容市貌明显改观。县城建设扩容提质，顺平、望都、容城等14个县(市)成功创建“省级园林县城”，安新、定兴、雄县荣获省人居环境进步奖。新农村建设有序推进，美丽乡村建设取得新进展，农民生产生活条件明显改善。2015年全市城镇化率达到46.65%，比2010年提高7.3个百分点。

生态环境逐步好转。重拳出击，铁腕治污，以前所未有的决心和力度推进生态环境治理，提高环境质量的基础条件正在改善。以“压煤、抑尘、控车、减排、迁企、增绿”为主线，开展大气污染治理十项攻坚行动，淘汰改造分散燃煤锅炉2532台，淘汰黄标车10.6万余辆，市区营运的870台公交车、3036辆出租车全部实现“油改气”。市区新增绿地面积1.37万亩，绿地率达到38.8%。全市完成植树造林总面积360万亩，森林覆盖率增加到26.7%，治理水土流失面积1073平方公里。2015年，市区空气质量达标天数为127天，比上年增加43天，重度及以上污染天数减少29天，PM2.5降低 17.1%，PM10降低22.3%。

改革开放深入推进。下大力气、啃硬骨头，进一步简政放权，不断释放改革红利，加大开放力度，激发企业活力。在全省率先公布行政权力“四份清单、一个标准”，取消和下放行政审批事项230项。取消和调整审批前置证明44项，进一步便利群众办事创业。积极探索新型市场监管模式，在全省率先组建市场监督管理局。深入推进商事制度改革，实施“三证合一、一照一码”登记制度，市场主体达到40万户，比“十一五”时期增加19万户，居全省第二。科技创新、财税体制改革稳步推进，金融、社会、土地等重点领域和关键环节改革取得积极进展，挂牌上市公司达到58家，比“十一五”增加46家，其中在境内外主板和新三板挂牌上市公司31家，位居全省第二。石保廊列入国家全面创新改革试验区，高碑店列为全国首批中小城市综合改革试点，白沟镇纳入第二批国家新型城镇化综合试点。市属经营性资产统一监管加快推进，国企改革重点难点问题取得突破性进展，遗留多年的蠡县农村信用社金融风险彻底化解，大世界商业城改造启动，依棉集团债务达成和解，公交管理体制改革圆满完成。坚持“引进来”、“走出去”并举，大力开展招商引资，累计利用外资25.4亿美元，比“十一五”增长25.1%，招商活动累计签约项目162项，总投资3784亿元。鼓励长城、英利等优势企业对外投资建厂，累计对外投资5.6亿美元政府工作报告。

人民生活持续提高。持续增加民生投入，不断扩大公共服务供给，共享发展成效显著。五年来市财政用于民生和社会事业支出1688.6亿元，是“十一五” 的2.6倍。城乡居民人均可支配收入分别达到23663元、10558元，比2010年分别增长57.3%、93.9%。城镇新增就业累计达到41万人。社会保障体系不断完善，城镇低保标准由每月283元提高到390元，农村低保标准由每年1400元提高到2300元，新农合和城镇居民医保财政补助标准均由120元提高到380元。实施扶贫攻坚，减少贫困人口72万人，提前一年完成“十二五”减贫任务。开工建设保障性住房16.4万套(户)，竣工8.75 万套(户)。九年义务教育完成率、高中阶段毛入学率分别达到94.5%、90%，比2010年分别提高了1.9和7.8个百分点。公共文化体系不断完善，文化产业发展势头良好。公共卫生和医疗服务体系实现城乡全覆盖。现代邮政业发展迅速，快递业务量首次突破一亿件大关。社会综合治理能力显著提升，社会稳定局面持续巩固。安全生产、食品药品安全形势总体平稳。审计、统计、质监、土地、供电、通讯、国防动员、民族宗教、新闻广电、外事侨务、人民防空、史志档案、气象地震、妇女儿童、老龄、残疾人等工作取得新成绩。

刚刚过去的2015年，是保定发展史上极不平凡、具有里程碑意义的一年。有四件事可以写入保定发展史册：第一件事是《京津冀协同发展规划纲要》的正式实施，把“推动京津保地区率先联动发展”纳入国家战略，开启了保定发展的光明未来;第二件事是市区部分行政区划调整和更名顺利完成，实现了人民群众大保定建设的多年夙愿;第三件事是津保铁路正式通车运营，圆了津保两地铁路直达的百年梦想;第四件事是经济总量突破3000亿元大关，保定正在向全省第一方阵迈进。此外，我们还成功举办了百家央企进河北、首届世界冀商大会保定投资推介会、首届白洋淀国际服装文化节、首届白洋淀国际创新论坛、第三届中国(高碑店)国际门窗博览会、第四届中国市场大会(白沟)等一系列重大活动，提高了我市的知名度和影响力，保定正在成为国内外瞩目的焦点。

各位代表!“十二五”时期我市经济社会发展取得的成就，是省委、省政府和市委科学决策、正确领导的结果，是全市广大干部群众团结协作、艰苦奋斗的结果。在此，我代表市人民政府，向全市人民，向人大代表、政协委员，向各民主党派、工商联、无党派人士和人民团体，向中央、省驻保单位，向驻保部队和武警官兵，向所有关心保定发展的国内外朋友，致以崇高的敬意和衷心的感谢!

各位代表!回顾五年来的发展历程，我们取得的成就来之不易，积累的经验更加珍贵。一是必须坚持跨越赶超发展，二是必须坚持低碳高端发展，三是必须坚持统筹协调发展，四是必须坚持改革开放发展，

五是必须坚持精准高效发展，六是必须坚持为民惠民发展。总结成绩和经验的同时，我们也清醒地认识到，经济社会发展中长期积累的深层矛盾和问题尚未根本解决，“十三五”期间依然面临着很大困难和挑战。一是产业层次较低，主导产业支撑力不强，新兴产业比重偏小，高端产业缺技术，低端产业缺成本竞争力，有效需求和有效供给不足并存，改革开放还需进一步加力。二是县域经济实力较弱，市县财政收入增幅收窄、刚性支出增多，财政收支矛盾突出。三是城镇化明显滞后，中心城市建设水平仍需提高。特别是有些市政工程进展缓慢，如白洋淀大道、乐凯大街南延、复兴路西延等项目因资金和征拆问题没有完成预期目标，改善城乡面貌、推进美丽乡村建设任重道远。四是资源环境约束逐年加大，环境保护和生态建设压力依然很大。特别是大气污染治理，尽管我们采取了很多措施，但对解决问题的复杂程度和基础条件的薄弱状况估计不足，治理效果与群众的切实感受和殷切期盼仍有很大差距，工作措施和工作力度还需要进一步加强，必须凝聚合力、超常发力、久久为功。五是民生和社会事业欠账较多，保障基本民生、兜住底线、实现精准脱贫任务十分艰巨。六是政府职能转变还不到位，一些地方和部门服务意识不强、行政效率不高，少数工作人员素质能力、工作作风、精神状态与新形势新任务的要求不相适应。对于这些问题，我们一定高度重视，坚持问题导向，坚决追究问责，采取有力举措，认真加以破解，不辱历史使命，不负人民重托。二、“十三五”时期的主要目标任务

“十三五”时期，是我市贯彻落实京津冀协同发展战略的黄金机遇期，是经济发展向中高端迈进的阶段转换期，是全面建成小康社会的战略决胜期，是生态文明建设的关键推进期，是全面深化改革的攻坚突破期，是社会民生事业发展的提质升级期。“夫天地之气，其会也有时;山川之灵，其发也有机。(引自明朝版《保定府志》)”保定正处于天时地利人和的黄金时点，我们绝不能错失前所未有的历史机遇，绝不能辜负人民群众的美好期盼，绝不能丢下身上的使命担当，必须焕发激情、燃烧激情，团结带领全市人民，凝心聚力，真抓实干，把“十三五”规划好、实施好，奋力开创我市经济社会发展新局面。

“十三五”时期我市经济社会发展的指导思想是：全面贯彻党的十八大及中央、省、市一系列会议精神，坚持“四个全面”战略布局，牢固树立“创新、协调、绿色、开放、共享”发展理念，高举发展、团结、奋斗旗帜，紧紧把握“协同发展、转型升级、又好又快”主基调，以推动京津保地区率先联动发展为方向，以全面建成小康社会为目标，大力实施协同发展、创新驱动、环境支撑三大主体战略，突出把城市做大、产业做强、生态做美、民生做富，积极发挥京津冀协同发展核心区功能，聚力打造区域性中心城市，全力建设京畿强市、善美保定，为全面建成小康社会、加快实现保定振兴而奋斗。

主要目标是：到“十三五”末，实现“一个确保、两个翻番、三个率先、四个提升”。“一个确保”，就是确保到2020年如期全面建成小康社会。“两个翻番”，就是生产总值比2010年翻一番以上，年均增长9%，城乡居民人均可支配收入比2010年翻一番以上。“三个率先”，就是在提高发展平衡性、包容性、可持续性的基础上，经济增长保持中高速，在京津冀区域率先增长;发展水平迈向中高端，质量效益在京津冀区域率先提升;生态环境显著好转，空气质量在京津冀区域率先改善。“四个提升”，就是科技创新支撑力显著提升，战略新兴产业占规模以上工业增加值比重高于全省平均水平;新型城镇化水平显著提升，常住人口城镇化率达到60%以上;产业结构的整体层次显著提升，主导产业支撑作用明显增强，现代服务业快速壮大，农业现代化取得突破进展;社会治理水平显著提升，社会治理体制机制不断创新，社会更加和谐稳定，社会文明达到新水平。

为确保全面建成小康社会，今后五年，我们将在“五个发展”上集中发力。

坚持创新发展，积极建设创新驱动发展示范市。把创新作为发展第一动力，充分发挥创新在经济发展中的引领作用，大幅提高科技进步贡献率，以创新重塑优势，让创新蔚然成风。以建设石保廊国家全面创新改革试验区和京南科技成果转化试验区为契机，以白洋淀科技城、高新区、保定·中关村创新中心、涿州国家农业科技园等为依托，面向世界建设国家创新驱动发展示范市。以产业链、资金链对接为突破口，促进京津保创新链深度融合。加快建立科技创新体系，推动科技与经济紧密结合，着力在汽车、新能源、生物医药、纺织服装、装备制造等重点产业，实施重大科技项目攻关。强化企业创新主体地位，培育壮大高新技术企业，打造“中国电谷”升级版。以大众创业、万众创新为抓手，搭建平台、完善服务、营造环境，建设创新创业生态体系，激发全社会创新创业热情。完善鼓励保护创新的体制机制，支持鼓励驻保高校、科研院所和企业实施创新创业教育改革，对接京津高端研发，实现保定高效转化，努力在承接京津科技成果转化上实现新突破，推动“保定制造”向“保定创造”迈进。加强创新人才队伍建设，加强知识产权保护，鼓励支持专利创制。扩大新产品和服务供给，以供给侧创新释放消费潜力，以消费结构升级带动产业转型提升。到2020年，全市研究和实验发展经费投入强度达到2.6%，科技创新体系基本建立，京津科技成果与我市产业需求有效对接，基本建成高端引领的京津冀协同创新共同体。

坚持协调发展，强力打造京津冀区域性中心城市。全面落实《京津冀协同发展规划纲要》，突出协同发展主战略，牢牢把握京津保率先联动发展方向，把协同发展贯穿“十三五”发展的各领域和全过程，在交通、生态、产业方面实现率先突破，在创新、开放、民生方面实现率先联动，全力打造首都功能疏解的支撑点、京津产业转移的承接地、外资进入京津乃至北方市场的桥头堡。优化区域发展布局，构建“一城三区两带”协同发展格局。加快京津保交通一体化进程，加强生态环境协同治理。壮大特色产业发展，提升园区建设水平，大力发展县域经济。完善城市管理，建设智慧城市。加快新型城镇化建设，促进城乡统筹发展，补齐农业农村短板。推动基础设施向农村延伸，构建均等化公共服务体系。加快农村经济发展，转变农业发展方式，推进美丽乡村建设，提升农民生活品质。到2020年，承接非首都功能疏解取得显著成效，京津保率先联动发展取得实质进展，区域性中心城市地位基本确立，经济社会、城乡区域协调发展，新型工业化、信息化、城镇化、农业现代化同步发展。

坚持绿色发展，持续建设绿色低碳宜居新区。牢固树立保护生态环境就是保护生产力、改善生态环境就是发展生产力的理念，更加自觉地推动绿色发展、循环发展、低碳发展，努力实现经济发展、生活富裕、生态良好。严格落实问责制度，持续强化工作举措，坚决打赢大气污染防治攻坚战。推进山区综合开发，努力把西部山区打造成绿水青山、金山银山。优化全流域水资源调度，推进水污染综合整治。全力推进白洋淀生态保护治理，加强西大洋、王快水库水源地保护。加快建设京津保城市间生态过渡带，统筹抓好山水林田湖生态保护和修复，创建协同发展生态示范区，争创国家森林城市。深入开展节能降碳，促进资源节约集约利用，构建循环经济体系，推动资源利用方式根本转变，推进低碳城市建设。到2020年，单位GDP能耗降低和主要污染物排放达到省定目标，森林覆盖率提高到35%，污染治理和生态修复实现重大突破，天蓝、地绿、水清、村美的生态环境基本展现。

坚持开放发展，不断增强发展内生动力。进一步解放思想、更新理念，着力破除体制机制障碍，以改革促开放，突破重点领域改革，完善提升开放体系，促进经济快速发展。继续坚持“引进来”与“走出去”相结合，拓展对内对外开放广度和深度，扩大招商引资范围，促进区域交流协作。创新外资利用方式，扩大对外贸易规模，加强开放载体建设，推进军民融合发展，大力发展开放型经济，全面提高对外开放水平。推进供给侧结构性改革，推动三次产业融合发展，打造现代产业体系升级版。加快发展生产性和生活性服务业，五年后服务业增加值占全市生产总值的比重达到45%。做大做强汽车、新能源、高端装备与智能制造三大先进制造业，培育壮大生物健康、新一代信息技术、新材料、节能环保等四大新兴产业，优化提升纺织服装、食品加工、新型建材三大传统优势产业。推进行政体制改革，持续简政放权。推进国资国企改革，提高国有经济活力和竞争力。推进财税金融价格体制改革，强化金融支撑实体经济作用。推进城镇化、农村改革，加快城乡统筹发展。推进教育、文化、体育、医疗卫生体制改革，最大程度满足人民的期盼。到2020年，开放合作的能力和水平大幅提升，开放型经济体系基本确立，重点领域和关键环节改革取得决定性成果，大开放格局全面形成。

坚持共享发展，全面建设幸福和谐保定。着力保障和改善民生，解决人民最关心最直接最现实的利益问题，让发展成果更多更公平惠及全市人民。拉开主战场，找准发力点，探索新模式，汇聚脱贫强大合力，坚决打赢精准脱贫攻坚战。把就业作为民生之本，促进更加充分更高质量就业。完善工资增长机制，努力实现居民收入与经济发展同步增长，着力提高居民收入水平。推动社会保险全覆盖，完善社会救助福利体系，推进民政事业大力发展，健全住房保障和供应体系，实现更加公平可持续的社会保障。加快发展人民满意教育，大力推进义务教育均衡发展，全面提高教育质量。积极发展卫生体育事业，全面提高人民健康水平。全面实施一对夫妇可生育两个孩子政策，积极应对人口老龄化。推进文化事业和文化产业繁荣发展，积极培育文化创意产业，努力建设文化强市。建立健全食品药品安全治理体系，加强和创新社会治理，深化“平安保定”建设，强化安全生产，构建全民共建共享的社会治理格局。到2020年，农村贫困人口全部脱贫，贫困县全部摘帽，人民群众生活质量、健康水平、居住环境明显改善，社会和谐稳定，人民生活幸福。

各位代表!“十三五”宏伟蓝图已经绘就，决胜全面建成小康社会时不我待。只要我们万众一心、奋力拼搏，扎实苦干、执着前行，“十三五”规划确立的各项目标就一定能够完成，重塑保定辉煌、找回大市尊严的美好愿望就一定能够实现!三、2016年重点工作

2016年是“十三五”开局之年、京津冀协同发展的集中发力之年，也是全市各项工作的深化改革年、攻坚落实年，做好今年的政府工作至关重要。今年经济社会发展主要目标是：生产总值增长8%，规模以上工业增加值增长7.5%，固定资产投资增长15%，一般公共预算收入可比增长9%，城乡居民人均可支配收入分别增长9.5%和10.5%，居民消费价格指数控制在103%左右，城镇登记失业率控制在4.5%以内，节能减排相关约束性指标完成省定目标。

今年我们将紧紧围绕京津保率先联动发展聚焦聚力，突出抓好七方面重点工作。

(一)聚焦协同发展，深度对接京津。把协同发展作为头等大事，紧盯重点产业园区和重大项目建设，抓好落地转化，承接疏解转移，加快与京津对接融合。

抓好专项规划。发挥规划引领作用，在京津冀协同发展战略总体框架下，围绕推进生态、交通、产业、环境、创新、开放、民生等领域率先突破，制定完善“十三五”专项规划，形成“十三五”总体规划、专项规划、年度计划相互支撑、层次分明、功能清晰的规划体系。做好与京津各领域专项规划对接，争取更多事项纳入国家、省大盘子。

推进多维合作。继续深化与京津对接，实现与北京城六区的战略合作全覆盖。瞄准中央国家机关、央企、知名企业、世界500强企业、学校医院、科研院所、大型市场，围绕科技创新、成果转化和产业转移开展合作，争取更多优质项目落户保定。加强与北京金融总部、天津金融创新运营示范区合作，促进京津金融主体在我市设立分支机构。加强与京津外商协会对接，推动外资企业向我市转移。

做强承接平台。着力打造高端设施配套、高端功能支撑、高端产业项目和人才聚集的精品园区。加快白洋淀科技城、保定·中关村创新中心、中关村丰台园满城分园、安国现代中药工业园区、涞源白石山中美科技国际创新园、京涞产业新城、英利科技生态小镇、中关村北服时尚产业创新园(容城)示范基地等承接平台建设。在徐水、高碑店、容城、涞源、望都、高阳等区域打造一批国别(地区)产业园。1至2年内，每个县(市、区)至少建设一个高标准的承接平台、至少引进一家大的战略合作者、至少有一个在全市乃至全省叫得响的对接合作项目。

加快项目落地。对省、市重点项目和近年来与京津签署的战略合作协议事项，明确责任领导和责任单位，全程跟踪服务，确保落地转化。重点推进长城汽车整车及零部件、涞水电子科技产业园、安国中药都、立中3D打印新材料产业园、高碑店国家建筑节能技术国际创新园、易县抽水蓄能电站等项目建设，加快同仁堂直隶中医医院、301医院涿州保障基地、保定轨道交通产业园建设。

(二)聚焦创新驱动，促进转型升级。把创新摆在发展全局核心位置，优化创新资源、激活创新主体，引领新供给、创造新需求、培育新动力，构建与京津协同发展的现代产业体系。

强化创新支撑。制定“十三五”科技创新专项规划，研究出台一批科技创新支持政策，启动高新技术产业三年倍增和科技型中小企业成长计划，厚植创新沃土。强化企业创新主体地位，鼓励企业加大研发投入，提高自主创新竞争力，培育更多科技创新型领军企业，新认定一批高新技术企业、创新型企业、工程技术中心和重点实验室。实施新兴产业“双创”三年行动计划和中小微企业扶持发展工程，推广众创、众包、众扶、众筹模式，建设京津保创新创业大街和中关村创新基地、保定 1898咖啡馆、保定科技金融创新中心等“双创”项目。每个县(市)建设一批回乡创业园，打造一批众创空间、创新工场。实施人才引进计划，开展“创业导师保定行”活动，搭建京津冀大学生创新创业平台，承接承办京津冀科技创新年会、论坛、峰会、科技展会，吸引海内外和京津优秀人才来保创业。

推动工业转型升级。深入推进制造业结构调整，促进制造业与信息化深度融合。实施好“中国制造2025”和“互联网+”行动计划，改造提升主导产业，推动汽车、新能源及输变电、纺织、食品、建材等产业向智能、高端、绿色、规模化发展。积极拓展新兴产业领域，大力培育新一代信息技术、新材料、节能环保、生物医药及高性能医疗器械产业快速发展。制定高端装备产业发展规划，大力推进智能制造，推动老工业基地改造，加快老企业退城进园进度。推进自主品牌建设，提高产品附加值和市场竞争力。继续推动“质量兴市”，加大质量监管力度，加强质量诚信体系建设。狠抓节能降耗，淘汰落后产能。

加快发展现代服务业。构建现代物流服务体系，培育大型物流企业，争创国家级现代物流创新发展城市试点。加快白沟“京津冀现代智慧商贸物流产城融合示范区”建设。加强与国内外物流名企合作，培育大型第三方物流企业，加强与知名电商合作，启动跨境电商平台建设，推动白沟箱包、蠡县皮毛、容城服装、曲阳石雕定瓷、安国中药、高阳纺织、雄县革塑、定兴绿色蔬菜、满城生活用纸等电子商务平台建设，做优做实“淘宝特色·中国保定馆”、“中国网库·电商谷”等一批地方电商网站，打造保定高端电商园区。发展科技服务业，推进技术开发及成果转移、科技信息及咨询、公共技术服务检验检测平台建设。高起点规划建设面向京津的养老服务业，承接京津养老转移，打造华银(天鹅湖)国际生态城等一批高品质养老小镇。发挥金融支撑助力作用，优化金融生态环境，推动各县(市)继续做大做强投融资平台，完成地方企业债发行工作。引导支持企业上市融资，引进一批重点金融机构，加快村镇银行设立进程。支持保定银行走出去，保定银行石家庄分行今年上半年开业。

今年9月，我市将承办全省首届旅游业发展大会，这是促进保定乃至全省旅游业整体跃升，吸引全球目光关注河北、聚焦保定，打造京津冀协同发展引爆点的一次盛会，必将成为全省产业转型的亮点、供给侧结构性改革的样板、又好又快发展的名片。以此为契机，全面加快推进涞源、涞水、易县旅游产业提档升级，整合旅游资源，推进景区创建，在全市建设一批省级旅游度假区、一批旅游强县、一批旅游名镇名村和有震撼力的新景区、新业态，加快建设首批国家级旅游业改革创新先行区。推动清西陵、白石山创建5A景区，抓好阜平、涞水国家旅游扶贫试验区建设，推进涞源、涞水、易县、安新创建“国家全域旅游示范区”。

加速推进农业现代化。全面落实今年中央一号文件，多措并举弥补农业农村发展短板。发展高效、优质、生态、品牌、安全农业，打造环京津1小时鲜活农产品物流基地和京津优质绿色农产品供应保障基地。抓好粮食生产核心区建设，全市粮食播种面积调减到1150万亩、粮食产能稳定在500万吨左右。做强蔬菜、果品、畜牧三大优势产业，发展渔业、中药材、食用菌等特色产业，加快保南苗木花卉基地建设，推进农产品质量标准认证，发展绿色产品、有机食品，打响保定品牌。推进环首都现代农业科技示范带、新发地物流园和涿州、白洋淀等农业科技园区建设，建设精品园区20个、乡镇园区200个，形成“一县一精品园区、一乡一特色园区”的发展格局。发挥多种形式农业适度规模经营引领作用，加快培育新型职业农民。壮大一批产业龙头，发展家庭农场、专业大户、农民合作社等新型经营主体。培育壮大新产业新业态，推进农村产业融合发展。按照“互联网+农业”思路，培育以电子商务为主的新型流通业态，加快构建“接二连三”的农业全产业链，建设农业强市。

(三)聚焦城乡统筹，加快城镇发展。立足打造全国新型城镇化和城乡统筹示范区，推进区域性中心城市建设，打好县城建设攻坚战，建设一批首都卫星城和京津冀“微中心”。加快产城融合、城乡互动，建设美丽乡村，提高新型城镇化发展水平。

加快重大基础设施建设。围绕把保定建成“全国性综合交通枢纽”，重点谋划推进“京津保城际铁路环线”和保忻、保沧等铁路建设，年内完善京石客运专线项目土地手续，力促廊涿、固保城际项目尽早开工。谋划与北京新机场的快速交通路网衔接工程，加快荣乌、曲港、太行山(西阜、涞曲)、津石等高速公路建设，构建“四纵四横四联两环”高速路网。加快打通连接京津的断头路、瓶颈路，推进国道112线曲村至张家口段、京原支线改造和张定线改造工程。加快深圳能源西北郊热电、涿州京能热电联产等项目建设，推进大唐保定热电厂九期、华润东北郊热电联产项目、大唐高碑店热电联产项目前期工作。推进液化天然气应急储备中心、市域燃气战略储备库、电动汽车充电站和交流充电桩等项目建设。力争启动1至2个天然气分布式能源电站示范工程。

加快中心城市建设。启动新一轮城市总体规划修编，推动“多规合一”。围绕打造区域性中心城市，推进中心城区东拓、西扩、南联、北延，拉大城市框架，做大城市规模，提升城市品位。着手谋划城市立交桥、高架桥、快速路、地铁和轻轨建设，启动双源无轨电车项目建设，打造立体化交通网络。完成白洋淀大道后续工程，推进乐凯大街南延，启动东风路西延，抓好西三环南延、隆兴路西延、北三环改造并西延，确保瑞祥大街北延、复兴路西延部分路段年底通车。谋划实施清苑、满城、徐水联接中心城区路网建设，推动新三区尽快融入主城区联动发展。注重地上地下统筹，加快城市供排水、污水处理、供热、供气、供电和垃圾处理等公共服务能力建设，开展城市地下综合管廊试点和海绵城市建设，谋划市区新建污水处理厂和配套管网项目。巩固国家园林城市成果，启动国家生态园林城市创建，谋划实施植物园、竞秀公园提升改造，推进立体绿化、屋顶绿化和林荫停车场建设，打造京石高速引线、七一路、南二环、东二环、长城南大街、天威西路、天鹅路、五四路和阳光大街9条景观大道。建立莲池区古城保护区，加快西大街业态整治，进一步彰显古城文化底蕴。推进东湖文化中心、西湖体育中心建设，关汉卿大剧院和市博物馆年底力争投入使用，启动建设高水准的图书馆、档案馆、城市规划展览馆和体育场馆。加快漕河综合治理，打造市区精品景观带。加速三大片区建设，年底前回迁安置房全部竣工;完成大世界、菊胡同棚户区改造阶段性任务，启动铁路四里营棚户区改造和西湖文体中心棚改集中安置区项目。持续推进市容环境综合整治，实现市区主次干道环卫清扫保洁市场化运作全覆盖，加强马路市场、占道经营及火车站、汽车站、学校、医院周边环境秩序重点管控。积极开展智慧城市建设，打造互联网宽带示范城市。

推动县城扩容提质。实施“小县大县城”战略，推进县城建设三年攻坚行动，打造一批京津保都市卫星城、一批微中心和一批美丽县城。着眼于世界级城市群建设，提高城市总体设计标准，对县城建设统一布局，形成有地域特色的城市风貌框架体系。深入推进“七个一”工程升级版，重点加快基础设施和公共服务设施建设，年内所有县城实现集中供水、供热或清洁能源供热、污水垃圾达标处理。涿州、涞水创建国家园林城市(县城)，高阳、蠡县、博野、涞源、曲阳、唐县、阜平等7县创建省级园林县城，确保年内实现省级园林县城全覆盖。以样板街打造为抓手，实施环境容貌专项整治，年内城区机械化清扫率达到57%以上。按照县域经济增比进位要求，突出产业特色，培育壮大产业园区，抓好新型工业化示范基地建设，每个县(市)集中支持一个主业突出、发展潜力较大的产业集群，确保县域经济主要指标增速高于全省平均水平。推进乡改镇、乡镇改办事处、村改居，提高户籍人口城镇化率。抓好白沟国家新型城镇化综合试点和高碑店全国中小城市综合改革试点，推进涿州松林店镇、定兴固城镇行政管理体制改革试点工作，培育一批各具特色的旅游名镇、工业小镇、商贸强镇。

推进美丽乡村建设。开展农村人居环境整治行动和美丽宜居乡村建设。按照“四美五改”要求，坚持连片开发，因地制宜、分类指导，掀起美丽乡村建设的热潮。对全市4470个未改造村庄，分类分批逐步推进。重点加快涞涞易、阜平省级示范区，顺平、曲阳、唐县、满城市级示范区和平原县县级示范区建设，集中开展民居改造、道路硬化、饮水安全等12个专项行动，发挥好重点村和示范片区的引领作用。在全市所有村庄集中开展清杂物、清残垣断壁和路障、清庭院、拆违章建筑和垃圾治理专项行动，建立垃圾处理长效机制，努力改变农村“脏乱差”面貌。

(四)聚焦改革开放，增强发展动力。啃硬骨头抓改革，破除藩篱促开放，释放深化改革新红利，增创扩大开放新优势。

推进供给侧结构性改革。落实好国家对实体经济减税减负、帮扶特困企业政策措施，综合施策降低实体经济制度性交易成本、社会保障成本、生产要素成本。继续取消下放一批市级审批事项，优化公共服务流程;推进实体和网上虚拟“中介超市”、网上审批服务平台建设;构建覆盖城乡的市、县、乡、村四级政务服务体系，打造“三级平台、两个代办”升级版。深化商事登记制度改革，探索电子化登记管理。深化财税金融体制改革，全面推开“营改增”改革，推进个人所得税和消费税改革，进一步实施减税降费政策。

深化重点领域改革。深化国资国企改革，加快推进市本级经营性国有资产集中统一监管，对市属国有企业实施分类改革和监管，推进食品行业国有企业改制重组。完善金融监管体系，加快商业银行发展和农村信用社综合改革。加快信用体系建设，初步建成社会信用体系数据库和共享平台。深化农村综合改革，推进农村产权制度改革，年内基本完成农村土地承包经营权确权登记颁证，建设市、县两级农村产权交易中心。开展农宅合作社试点，促进闲置农宅流转利用。推进供销社综合改革。统筹推进医药卫生、食药监管和价格机制改革，完成市县公车改革。

加大招商引资力度。抓好招商项目的谋划、筛选和储备，积极组织参加全国知名民企助推河北协同发展大会、唐山中东欧招商大会等重大招商活动。加强与央企、国内外500强大企业集团战略合作，促成一批外资超亿美元、内资超10亿元项目签约落地。以开发区建设为载体，深化开发区体制机制改革，推广政府推动、企业经营、市场运作、多元投入的经营模式，推动开发区转型升级创新发展，打造一批战略性开放合作平台。

培育对外贸易优势。支持汽车及零部件、新能源等优势产业参与国际产能合作和境外并购，培育一批具有核心竞争力的跨国企业。筹建保定综合保税区，推进本地电子口岸建设，完善内陆港功能。落实外贸稳增长政策措施，加大重点出口企业引导扶持力度，加强出口基地建设，推动英利、巨力、光为等新能源企业光伏产品出口，加快外贸转型升级。加强与中东欧和“一带一路”沿线国家的合作，开拓东盟、非洲、南美等新兴市场。

(五)聚焦环境支撑，改善发展环境。持续打好优化环境“组合拳”，着力解决软、硬环境问题，增强我市发展的环境魅力。

全力攻坚大气治理。坚持科学治霾、依法治污，加强与京津联防联控，继续深入实施大气污染防治攻坚行动，强化减煤、抑尘、控车等重点措施，促进大气质量明显改善。严控煤炭消费总量，实施城区集中供热攻坚，加快淘汰35蒸吨以下燃煤锅炉，全面完成三环以内农村煤改气工作。全面治理扬尘污染，实施“过境大车市区限行、主要道路两侧绿化提升和支路硬化抑尘”三项行动，严格整治工地、道路和矿山扬尘。严控机动车污染排放，坚决淘汰黄标车。加强挥发性有机物治理，严防“土小”企业反弹。加快地热等清洁能源开发利用，狠抓秸秆禁烧和综合利用。

改善修复生态环境。推进水污染防治行动计划，深入实施白洋淀及流域综合治理，开展重污染河流综合整治、府河污水库和黑臭水体治理，严格饮用水源地保护，加强地下水超采综合治理。完成南水北调地表水厂及配水管网建设，实施引黄入冀补淀工程。综合防治农业面源污染，改善土壤环境质量。加快山体修复和矿山生态整治，加强湿地保护。加快太行山绿化和京津保平原区生态过渡带建设，抓好封山育林、三北防护林、退耕还林(湿)、京保合作造林等生态项目建设，全年完成造林70万亩。实行网格化环境监测预警体系，实现市区主要路段、重点企业、重点区域污染监控全覆盖，依法严惩破坏生态环境的违法犯罪行为。

优化提升发展环境。推动率先联动发展，迫切需要我们全方位改善软环境。牢固树立群众至上、企业至上、服务至上的理念，建立市、县两级每月一次“为企业服务日”制度，全面叫响“来到保定，一切搞定”，发扬“马上就办、办就办好”的作风，坚决查处不作为、乱作为、慢作为等破坏环境的人和事，全力营造开放包容、高效务实的发展环境，吸引更多企业到保定落户，使更多京津高端要素向保定聚集。

打造和谐社会环境。严格落实安全生产责任制，深入开展重点行业领域安全生产专项整治，扎实做好隐患排查治理工作，强力推进安全生产标准化建设，坚决遏制重特大事故发生。强化食品药品安全监管，严格责任追究制度，推进行业诚信体系建设，持续开展食药打假专项整治行动。严厉打击非法金融活动，防范和化解金融风险。坚持用法治思维方式化解社会矛盾，高度关注民间借贷、土地征迁、企业改制、环境治理中的矛盾和问题。深化平安城市建设，常态化推进严打整治，重点打击涉枪涉爆、两抢一盗等违法犯罪活动，保持社会和谐稳定。

(六)聚焦精准脱贫，促进民生改善。“民之所呼，政之所向。”打好脱贫攻坚战，倾力保障改善民生，抓好“五保一创”，实施一批民心工程，办好一批民生实事，让群众有更多获得感。

推进精准脱贫攻坚。以9个贫困县为主战场，全面落实“六个精准”要求，对每个贫困家庭做到“六个确保”：确保危房都能得到修缮，确保家庭成员都能吃得饱、穿得暖，确保孩子有学上，确保家庭劳动力有活干，确保病人能够得到医疗救助，确保留守老人、留守妇女、留守儿童都能得到足够的社会关爱。按照“五个一批”要求，持续推进钱袋子、挪穷窝、聚宝盆、拔穷根、兜底线精准扶贫五大工程，确保全年脱贫17万人。把产业扶贫作为重点，用好金融扶贫政策，加快培育脱贫产业，推进荒山综合开发，引导贫困户增加收入。与推进工业化、城镇化、农业产业化有机结合，启动新一轮易地扶贫搬迁工程，打好“十三五”脱贫攻坚“当头炮”。充分发挥行业扶贫、专项扶贫、社会扶贫作用，加大财政资金投入。支持阜平打造精准脱贫示范县、涞水打造旅游扶贫示范县，推进政策性金融扶贫试验示范区建设。

强化就业和社会保障。全力扩大就业，继续实施就业优先战略和更加积极的就业政策，突出高校毕业生、城镇困难人员、退伍军人等重点群体，加大创业就业政策帮扶力度。落实失业保险金援企稳岗政策，稳定就业岗位。强化社会保障，完善城镇职工和城乡居民基本养老保险制度。完善新农合大病保险制度，实现大病保险全覆盖。完善社会救助体系，全面开展重特大疾病医疗救助。推进医疗保险京津冀区域内就医直接结算，加快社会保障“一卡通”建设。

加快社会事业发展。支持鼓励社会资本参与发展社会事业。深入推进健康保定建设，加强与京津医疗卫生联动协作，发展中医药事业，提高医疗卫生服务水平。促进教育公平，合理配置教育资源，加快推进教育领域三个“三年行动计划”，新开工山区寄宿制学校10所，建成县城义务教育学校31所、市区义务教育学校7 所，维修改造校舍5.2万平米。实施文体惠民，加快数字文物文化产业园、市区重点文物修缮工程、军校广场改造升级、长城保护工程等重点项目建设。加强国防教育和国防后备力量建设，推进双拥共建，促进军民融合深度发展。维护民族团结，促进宗教和谐。做好新闻出版广电、外事侨务、气象地震、防灾减灾、邮政通信、史志档案和老龄、妇女儿童、残疾人等工作。

办好惠民利民实事。去年我市确定的65件惠民事项基本完成。今年，我们本着群众关切、统筹协调、务实求真、量力而行的原则，计划实施社会保障、教育提升、卫生健康、扶贫济困、畅通工程、住房保障、便民服务、文化生活等8个方面55项惠民利民实事。对此，我们已经明确责任要求，一定采取严实措施，加强督导考核，确保把实事办实、把好事办好。

(七)聚焦效能提升，加强自身建设。推动保定大发展、大跨越，各级政府责任重大、使命光荣。我们一定以改革创新的开拓意识、昂扬向上的最佳状态，朝夕勤勉，永不懈怠，不断开创政府各项工作新局面，绝不辜负全市人民的厚望。

大胆解放思想，提高引领发展的能力。率先联动发展，必须率先解放思想。深化改革需要创新，攻坚克难需要突破，所有这些都需要我们打开思想解放的阀门，担负起新的发展重任。我们一定勤于学习、善于思考，适应新常态，把握新要求，提高把握规律能力，自觉增强工作本领;一定关注大势、把握形势，顺应大潮流，胸怀大格局，用新理念创造性地推动工作;一定勇于担当、敢为人先，不讲这不行、那不行，只说怎么行、怎么成，创造宽松环境，为市场主体放权松绑，为跨越发展助力加油。

严格依法行政，维护社会公平正义。坚持法定职责必须为、法无授权不可为。认真执行人大及其常委会的决议决定，依法接受法律监督和工作监督，主动接受人民政协的民主监督，认真办理人大代表建议和政协提案。建立社情民意反映制度、重大事项社会公示和社会听证制度，完善决策咨询制度，加强新型智库建设，积极发挥政府法律顾问和政府参事作用，做到重大事项事前充分论证，不断提升科学决策水平。注重提高各级干部的法治意识，严格公正文明执法，强化执法监督，加快建设法治政府，让政府工作在阳光下运行。

切实转变作风，做到重实干、讲认真、提效能。严格执行“八项规定”，坚决纠正“四风”，着力解决怠政懒政、不作为慢作为乱作为等问题。把夙兴夜寐、激情工作作为常态，把鞠躬尽瘁、殚精竭虑贯穿始终，把中央省市各项决策部署贯彻好、督导落实好。对重点难点工作一件一件地抓，一个环节一个环节地突破，一个节点一个节点地推进，铆足干劲把钉子钉到底。“动员千遍，不如问责一次。”今后，对各项工作都要严督实查，健全问责和激励机制，确保重大决策部署落地生效。

坚持清廉从政，建设百姓信赖的政府。坚持把纪律和规矩挺在前面，认真贯彻执行新修订的《廉洁自律准则》和《纪律处分条例》。对手中的权力心存敬畏，追求道德高线、严守廉洁底线、不踩法律红线，严以用权谋发展，严以用权为百姓，严以用权守正道。加强权力运行制约监督，加大违纪违法案件查办力度，严肃查处发生在群众身边的腐败问题，做到有腐必惩、有贪必肃，努力实现干部清正、政府清廉、政治清明。

各位代表!京津保率先联动发展的光明前景振奋人心，京畿强市、善美保定建设的美好愿景激励人心，如期全面建成小康社会的宏伟蓝图鼓舞人心!让我们紧密团结在以习近平同志为总书记的党中央周围，在省委、省政府和市委的正确领导下，团结带领全市人民，勠力同心，奋发作为，努力开创保定发展新局面，共同谱写保定振兴新篇章!
